# Foot Drop Caused by an Intraneural Ganglion Cyst: A Case Report

**DOI:** 10.7759/cureus.101336

**Published:** 2026-01-12

**Authors:** Abdullah Alhussein, Mohammed AlOthman, Nouf M Althobaiti, Sultan Alreshood, Sherif Elwatidy

**Affiliations:** 1 Neurosurgery, King Salman bin Abdulaziz Medical city, Madinah, SAU; 2 Neurosurgery, King Saud Medical City, Riyadh, SAU; 3 Neurological Surgery, King Khalid University Hospital, Riyadh, SAU; 4 Neurosurgery, King Saud University, Riyadh, SAU

**Keywords:** acute foot drop, common peroneal nerve, foot drop, ganglion cyst, intraneural ganglion cyst

## Abstract

Foot drop (FD) refers to a clinical picture characterized by the inability to lift the foot against gravity due to weakness of the dorsiflexor muscles. Patients with FD typically present with an abnormal gait marked by exaggerated flexion of the knee and hip joints, along with internal rotation of the foot, which may increase the risk of falls and injuries. Intraneural ganglion cysts are benign, fluid-filled lesions located within the epineurium of peripheral nerves. We report the case of a 22-year-old male who presented with right lower limb pain and numbness and later developed acute weakness of the right foot, resulting in complete FD that persisted for four months. He denied any history of trauma and was otherwise medically healthy.

On examination, there was muscle wasting in the anterior and lateral compartments of the right leg, complete FD with 0/5 power in dorsiflexion and eversion, and reduced sensation along the distribution of the right common peroneal nerve (CPN). Local examination revealed a palpable and tender swelling around the neck of the right fibula. Plain X-rays revealed no abnormalities; however, MRI demonstrated a small cystic soft tissue lesion located posterolateral to the proximal fibular head, measuring 5 x 1.5 x 2.5 cm. Surgical exploration of the right CPN was performed with neurolysis and excision of the lesion. Histopathological examination confirmed the diagnosis of a ganglion cyst.

Postoperatively, the patient had an uneventful recovery, and after two weeks of physical and occupational therapy, he reported improvement in pain and numbness, although motor function remained unchanged at that time. The association between FD and ganglion cysts is a rare pathology; however, it should be included in the differential diagnosis of FD, particularly in the absence of trauma.

## Introduction

Foot drop (FD) is characterized by an inability to dorsiflex the foot due to weakness of the dorsiflexor muscles, most often resulting from peroneal nerve pathology. This condition may be either unilateral or bilateral, depending on the location of the pathology, which can involve the central or peripheral nervous system or the dorsiflexor muscles [1]. Patients with FD often exhibit a characteristic gait, marked by hyperflexion of the knee and hip joints, as well as internal rotation of the foot [2], which significantly increases the risk of falls and injuries [3].

Intraneural ganglion cysts are benign, fluid-filled lesions located within the epineurium of peripheral nerves [4] and are most commonly observed in middle-aged males [5]. Peroneal nerve ganglion cysts are a recognized cause of FD and are often associated with sensory deficits. MRI, nerve conduction studies (NCS), and electromyography (EMG) are key diagnostic tools for detecting these lesions and differentiating ganglion cysts from other space-occupying pathologies around the peroneal nerve. This report describes a rare condition, as intraneural peroneal nerve ganglion cysts represent an uncommon cause of foot drop, presented in a 22-year-old male with a four-month history of non-traumatic FD and no significant medical history.

## Case presentation

**Figure 1:**
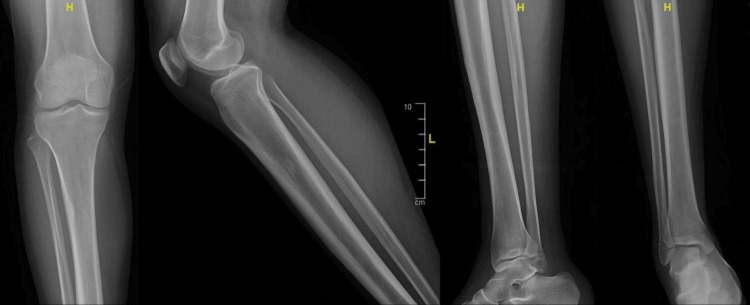
Radiograph showing no focal bony or joint abnormalities and no evidence of a fracture

A 22-year-old male presented with back and right lower limb pain accompanied by numbness and acute weakness in the right foot, resulting in FD for four months. He reported foot inversion, inability to dorsiflex, and numbness over the dorsal aspect of the foot upon waking. The patient was otherwise medically unremarkable and denied any history of trauma. On examination, significant muscle wasting was noted in the anterior and lateral compartments of the right leg, along with complete FD evidenced by 0/5 strength in dorsiflexion and eversion, and decreased sensation in the distribution of the right common peroneal nerve (CPN). A localized, painful swelling was palpable around the neck of the right fibula along the CPN course, with a positive Tinel’s sign. Radiographs showed no abnormalities (Figure 1).

**Figure 2:**
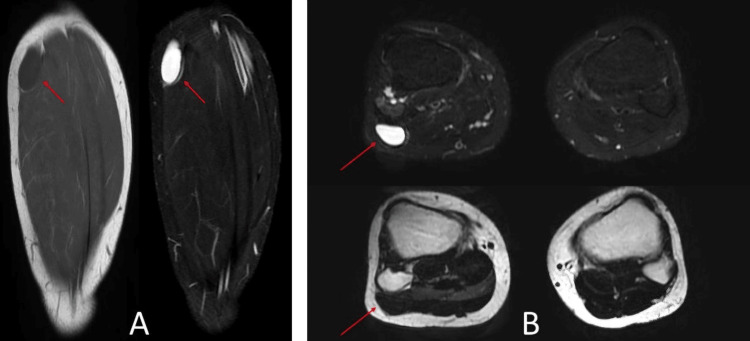
MRI findings Multi-planar multi-sequential MRI with contrast. (A) Coronal MRI of the right leg showing the lesion (red arrow). (B) Axial MRI of both legs with the lesion indicated by the red arrow MRI: magnetic resonance imaging

MRI with gadolinium contrast enhancement revealed a cystic lesion located posterolateral to the proximal fibular head, measuring 5 × 1.5 × 2.5 cm, with signal characteristics suggestive of a ganglion cyst or schwannoma (Figures 2A, 2B). Nerve conduction studies and electromyography demonstrated findings consistent with subacute to chronic axonal neuropathy, based on reduced amplitudes and denervation changes, affecting the tibialis anterior muscle.

**Figure 3:**
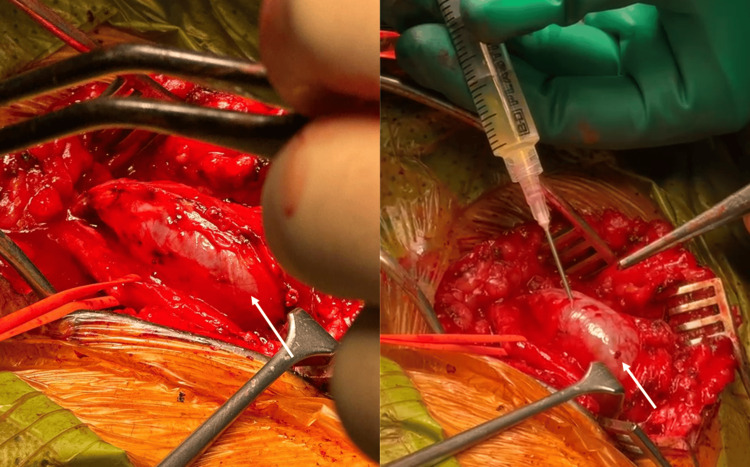
Intraoperative image showing needle aspiration of the cystic lesion (white arrow)

The patient underwent surgical exploration of the right CPN under general anesthesia, including neurolysis and excision of the cystic lesion. An S-shaped incision was made, revealing a large cystic lesion extending into the epineurium. The proximal and distal ends of the CPN were exposed, and the superficial peroneal nerve was separated from the lesion. Needle aspiration was performed to deflate the cyst (Figure 3), followed by complete excision. No intraoperative neuromonitoring was used. The postoperative course was uneventful, and physical and occupational therapy were initiated during the first postoperative week. Two weeks later, the patient reported improvement in pain and numbness in the right leg, while motor function remained unchanged, as expected at this stage.

## Discussion

This report highlights an uncommon cause of non-traumatic FD resulting from an intraneural ganglion cyst of the CPN. The diagnosis was established based on the characteristic clinical presentation, imaging findings, and electrophysiological evidence of nerve involvement, with early symptomatic improvement following surgical management. The presence of ganglion cysts is clinically evidenced by pain and numbness in the distribution of the affected nerve, resulting from nerve irritation. In cases of peroneal nerve ganglion cysts, FD develops due to muscle weakness, primarily involving the extensor digitorum brevis and tibialis anterior muscles [6].

Sensory testing often reveals reduced or absent sensation in the distribution of the affected nerve [6], while Tinel's sign is typically positive along the peroneal nerve. FD is a complex condition requiring a multidisciplinary approach for appropriate evaluation and treatment [7]. Numerous theories have been proposed to explain the underlying pathophysiology of intraneural ganglion cysts [8-10]. Among these, the synovial theory described by Spinner et al. [11] is the most widely accepted. This theory posits that endoneural ganglion cysts originate from a defect in the joint capsule, leading to the extravasation of synovial fluid into the subepineural space, resulting in cyst formation. This hypothesis is supported in the literature [12].

NCS and EMG play an important role in localizing the site of pathology and characterizing the nature of nerve injury. In the present case, electrophysiological findings were consistent with subacute to chronic axonal neuropathy, correlating with the clinical duration and supporting the diagnosis of a compressive peripheral nerve lesion. Differential diagnoses include L5 root compression and nerve sheath tumors, such as schwannomas. Radiographs are not definitive for diagnosing ganglion cysts, but are useful for ruling out conditions such as bone anomalies, fractures, and degenerative lumbar spine disease. Ultrasonography helps differentiate solid masses from cystic lesions, with solid masses appearing hypoechoic and cystic lesions as well-circumscribed anechoic masses [13-16].

Although ultrasonography is a cost-effective and noninvasive imaging modality, it lacks the sensitivity to reliably distinguish ganglion cysts from other nerve sheath tumors [17]. MRI is the preferred imaging modality for diagnosing ganglion cysts, offering superior sensitivity compared to other methods. However, differentiation from other nerve sheath tumors may remain challenging. Classically, ganglion cysts exhibit low signal intensity on T1-weighted images and high signal intensity on T2-weighted images [18], consistent with the findings in the present case.

The current gold standard for treatment is surgical removal of the lesion. While ultrasound-guided aspiration has been described as a less invasive option, it does not address the underlying synovial connection and is associated with a higher risk of recurrence. The prognosis for peroneal nerve palsy caused by ganglion cyst compression is generally excellent, although neurological recovery may take one to two years and can be either complete or partial [6]. Articular branch ligation is critical to prevent recurrence, as it eliminates the source of synovial fluid propagation, a principle emphasized in the literature [1,18-20].

This study has certain limitations. As a single case report, the findings cannot be generalized; additionally, the follow-up duration was relatively short. Nevertheless, this report underscores the importance of considering intraneural ganglion cysts in the differential diagnosis of foot drop, particularly in patients without a history of trauma.

## Conclusions

The coexistence of foot drop (FD) and ganglion cysts is an uncommon condition, but it should be considered in the differential diagnosis of FD, especially in non-traumatic cases. Diagnosis can be facilitated by MRI, ultrasound, and electrophysiological tests, with careful consideration given to ruling out L5 root compression and nerve sheath tumors. Surgical excision remains the standard treatment of choice. Overall, the prognosis is favorable, although recovery might span several months to years.
